# The evolutionary dilemma of broad-spectrum insecticides

**DOI:** 10.1093/biosci/biaf074

**Published:** 2025-06-16

**Authors:** Scott Glaberman, Kyle Spatz, John K Colbourne

**Affiliations:** Centre for Environmental Research and Justice, in the School of Biosciences at the University of Birmingham, in Birmingham, England, in the United Kingdom; Department of Environmental Science and Policy at George Mason University, in Fairfax, Virginia, in the United States; Centre for Environmental Research and Justice, in the School of Biosciences at the University of Birmingham, in Birmingham, England, in the United Kingdom

Insects are declining at an alarming rate worldwide, driven by multiple factors, including the use of agrochemicals (Goulson et al. 2015). Current pesticide safety assessments often underestimate their ecological risks, particularly to pollinators and other beneficial insects, because they rely heavily on short-term mortality tests conducted in a limited number of surrogate species, most often honeybees (Fisher et al. 2023). Moreover, new research using the model species *Drosophila melanogaster* has shown that field-realistic concentrations of many agrochemicals disrupt insect behavior, development, and reproduction, threatening long-term survival across insect populations (Gandara et al. 2024). These effects extend across multiple insect classes, highlighting the urgent need to assess risks at the level of entire arthropod communities.

## The evolutionary dilemma

**Figure 1.**
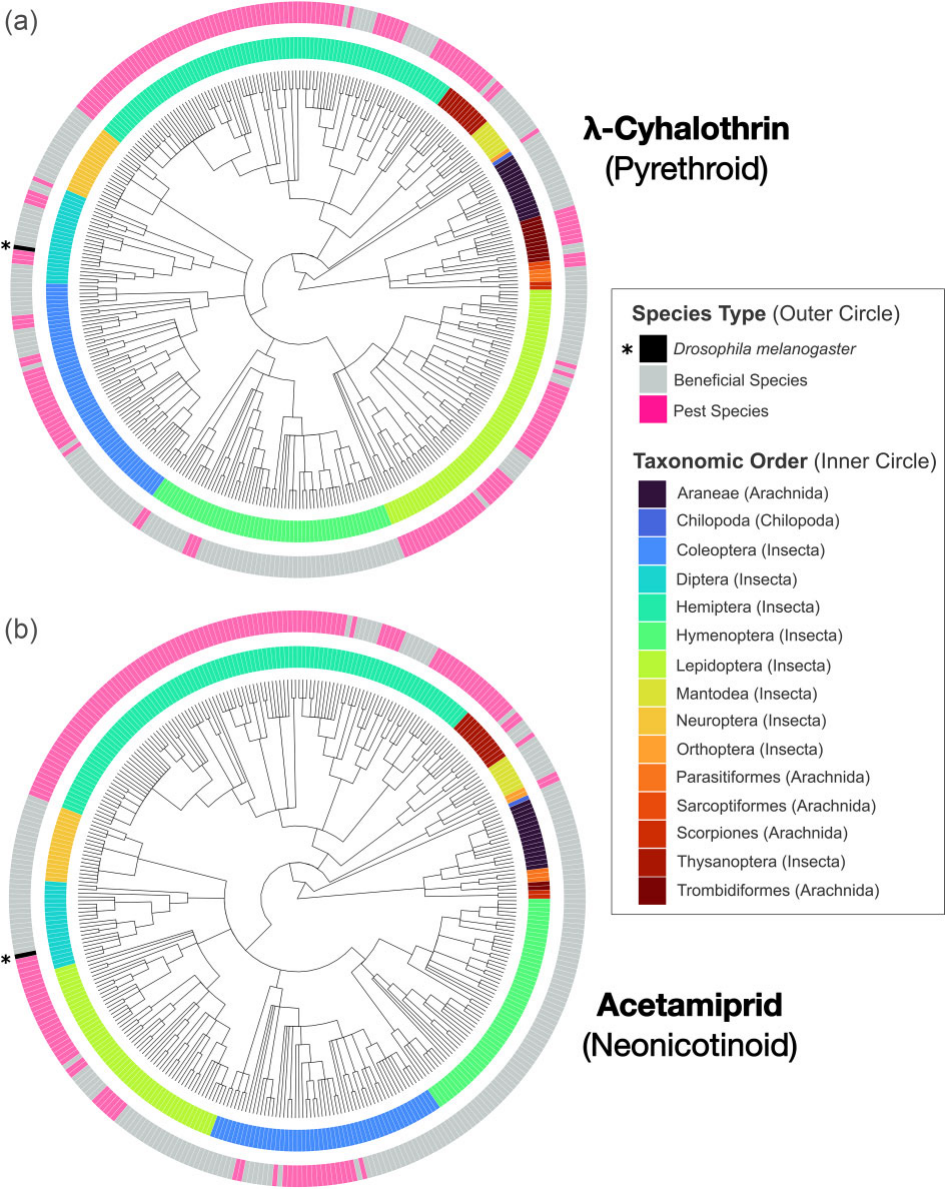
Phylogenetic trees showing evolutionary relationships among the model organism *Drosophila melanogaster* (black with asterisk), nontarget beneficial arthropods (gray), and pest species (pink) exposed to two widely used broad-spectrum insecticides: (a) λ-Cyhalothrin (pyrethroid) and (b) Acetamiprid (neonicotinoid). The inner color ring indicates taxonomic order. Pesticide associations are based on California Department of Pesticide Regulation data (www.cdpr.ca.gov), with pests identified from registered product labels. Beneficial species, including pollinators, predators, and parasitoids, were compiled from reports by the National Academies of Sciences (National Research Council 2007) and the University of California (Flint and Dreistadt 1998). These trees illustrate how pest and beneficial taxa are interwoven across the arthropod phylogeny, emphasizing the concept of toxicity by descent—that phylogenetic proximity contributes to shared vulnerability across target and nontarget species (Colbourne et al. 2022). This framework can inform ecological risk assessment across agroecosystems.

To fully grasp the implications of these findings, it is essential to adopt an evolutionary perspective. Pest species targeted by agrochemicals, as listed on their product labels, share close evolutionary relationships with model organisms such as *Drosophila* and beneficial species—such as pollinators, natural predators, and parasitoids—that are vital to ecosystem services (figure1). This evolutionary overlap creates a central dilemma: broad-spectrum insecticides—such as diamides, neonicotinoids, organophosphates, carbamates, and pyrethroids—are designed to target a wide array of pests but can similarly harm nontarget species through toxicity by descent (Colbourne et al. 2022). Physiological traits, such as chemical sensitivity, are often conserved across related species, leaving beneficial species just as susceptible as pests (Spurgeon et al. 2020). Following the precautionary principle, we should assume that beneficial species are equally susceptible unless demonstrated otherwise. Similarly, effects observed in model organisms such as *Drosophila* are likely to occur in closely related beneficial species. This is particularly concerning for pollinators, which are not only essential for ecosystem services but are also highly attracted to popular crops such as almonds, alfalfa, and apples, where these insecticides are often used (USDA 2014).

## Toward evolutionary solutions

**Figure 2.**
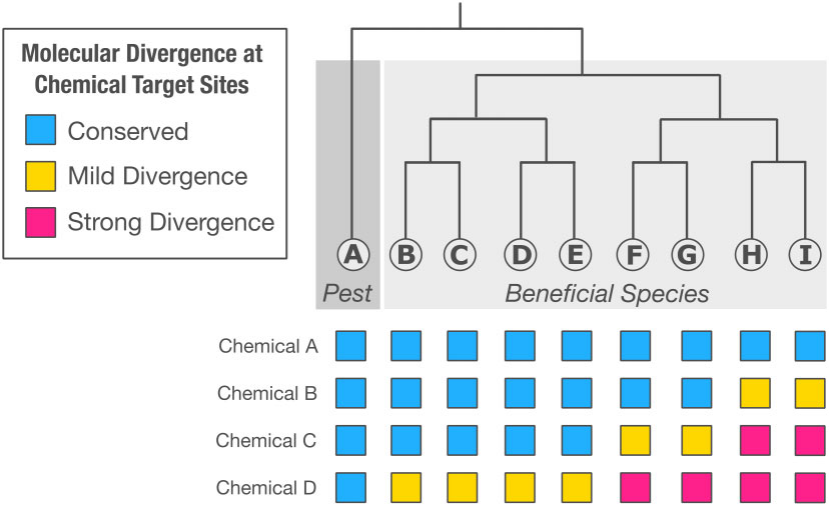
*Hypothetical example illustrating* phylogenetic relationships among a pest species (a) and beneficial species (b–i), with molecular divergence at pesticide target sites for four fictional chemicals (a–d). The color coding reflects the degree of divergence from the pest at each chemical's target site: blue, conserved; yellow, mild divergence; pink, strong divergence. Although conserved sequences suggest a low likelihood of differential susceptibility, even small molecular changes (e.g., a single amino acid substitution) can result in functional differences. Therefore, molecular divergence increases the potential for altered susceptibility. Evolution provides a context for interpreting divergence patterns, which can help prioritize species for further study or protection. A similar conceptual framework was recently used for bees (Bass et al. 2024).

Just as evolution illuminates this challenge, it can also be part of the solution. Emerging research shows that chemical susceptibility is, in part, due to differences in the molecular targets of agrochemicals (figure2; LaLone et al. 2021). Many of the best-characterized examples come from the insecticide resistance literature. For instance, mutations in voltage-gated sodium channels confer knockdown resistance to pyrethroids in both agricultural pests and disease vectors (Soderlund and Knipple 2003), as well as in nontarget species such as *Hyalella azteca*, where distinct point mutations have independently evolved across clades (Weston et al. 2013). Benzoylurea resistance in Lepidoptera has been linked to mutations in the chitin synthase gene (Van Leeuwen et al. 2012, Demaeght et al. 2014, Douris et al. 2016), and organophosphate resistance in mosquitoes is associated with single-nucleotide changes in the ace-1 gene encoding acetylcholinesterase (Weill et al. 2004). Similarly, variation in ryanodine receptor genes underlies resistance to diamides in *Plutella* and other species (Troczka et al. 2012). Cytochrome P450 enzymes of the CYP9Q subfamily enable honeybees to metabolize certain neonicotinoids, whereas these enzymes are absent or divergent in more sensitive pollinators such as bumble bees and solitary bees (Bass et al. 2024). Structural modeling of the ecdysone receptor in *Chrysoperla carnea*—a beneficial predatory insect—revealed binding site differences that prevent activation by the lepidopteran-targeted insecticide tebufenozide (Zotti et al. 2012). These cases illustrate how molecular divergence across the phylogeny can help identify species for further scrutiny or protection. Comparable approaches have also been applied in vertebrate toxicology to predict cross-species susceptibility on the basis of receptor similarity (LaLone et al. 2023). This same molecular precision can guide industry in selecting chemicals that avoid vulnerable nontargets.

Of course, not all agrochemical effects are mediated through known, conserved targets. Some chemicals, such as DDT, can cause unexpected off-target effects—such as thinning bird eggshells—through mechanisms not easily predicted by evolutionary proximity. For these cases, broad toxicity screening and emerging tools such as high-throughput assays, multiomics, and organ-on-a-chip systems are essential (Stucki et al. 2022). Our evolutionary framework is not a replacement for broad screening but a complement. When a chemical's mode of action is known and involves a conserved molecular target, evolutionary relationships can help anticipate which species are likely to be affected.

Evolution reveals a critical blind spot in agrochemical evaluation: shared ancestry increases the potential for shared susceptibility. Although broad toxicity screens remain essential, integrating phylogenetic and molecular insights can enable smarter prioritization, safer chemical design, and more predictive risk assessments. As insect populations continue to decline, evolutionary thinking offers a powerful step toward developing more targeted and ecologically informed chemical safety strategies.

## Author Biography

Scott Glaberman (s.glaberman@bham.ac.uk) and John K. Colbourne are affiliated with the Centre for Environmental Research and Justice, in the School of Biosciences at the University of Birmingham, in Birmingham, England, in the United Kingdom. Kyle Spatz is affiliated with the Department of Environmental Science and Policy at George Mason University, in Fairfax, Virginia, in the United States.
